# Inter-study reproducibility of interleaved spiral phase velocity mapping of renal artery haemodynamics

**DOI:** 10.1186/1532-429X-17-S1-Q39

**Published:** 2015-02-03

**Authors:** Jennifer Keegan, Hitesh Patel, Robin Simpson, Raad Mohiaddin, David Firmin

**Affiliations:** 1 Royal Brompton Hospital, London, UK; 2 Radiological Physics, University of Freiburg, Freiburg, Germany

## Background

Renal resistive index (RI) and pulsatility index (PI) are reliable measures of downstream renal resistance which correlate with the severity of renal disease. To date, MR phase velocity mapping studies have lacked the temporal resolution required to determine these pulsatility parameters. Our aim is to develop a high temporal resolution breath-hold spiral phase velocity mapping technique for assessment of the temporal flow patterns in renal arteries and to determine inter-study reproducibility.

## Methods

An interleaved spiral phase velocity mapping sequence was developed on a 3T Skyra scanner (Siemens) using 1-1 water excitation and with full k-space coverage in 8 spiral interleaves of 12 ms duration. Phase map subtraction of datasets with symmetric bi-polar velocity encoding gradients resulted in velocity maps with a through-plane phase sensitivity of +/- 150cm/s. Retrospectively ECG gated data were acquired in a 17 cardiac cycle breath-hold (includes 1 dummy cycle) with a spatial resolution of 1.4 x 1.4 mm (reconstructed to 0.7 x 0.7 mm) and a repeat time of 19 ms. Renal artery velocity maps were acquired in 10 healthy volunteers (10 left and 10 right arteries). Data were acquired in each of two separate scanning sessions with the volunteer leaving the scanner between sessions. For each acquisition (40 in total), RI and PI were calculated by 2 observers as follows:

RI = (PSV - MDV) / PSV

PI = (PSV - MDV) / MV

where PSV = peak systolic velocity, MDV = minimum diastolic velocity and MV = mean velocity through the cardiac cycle

Background phase errors were determined from (i) a large stationary phantom acquisition and (ii) by fitting a background phase map to user defined stationary points in the inter-vertebral disks. Inter-observer and inter-study reproducibility of RI, PI and renal artery blood flow (RABF) per kidney were determined as the mean (+/- standard deviation) of the paired differences between observers and between scanning sessions and by the intraclass correlation coefficient (ICC).

## Results

**Figure 1:**
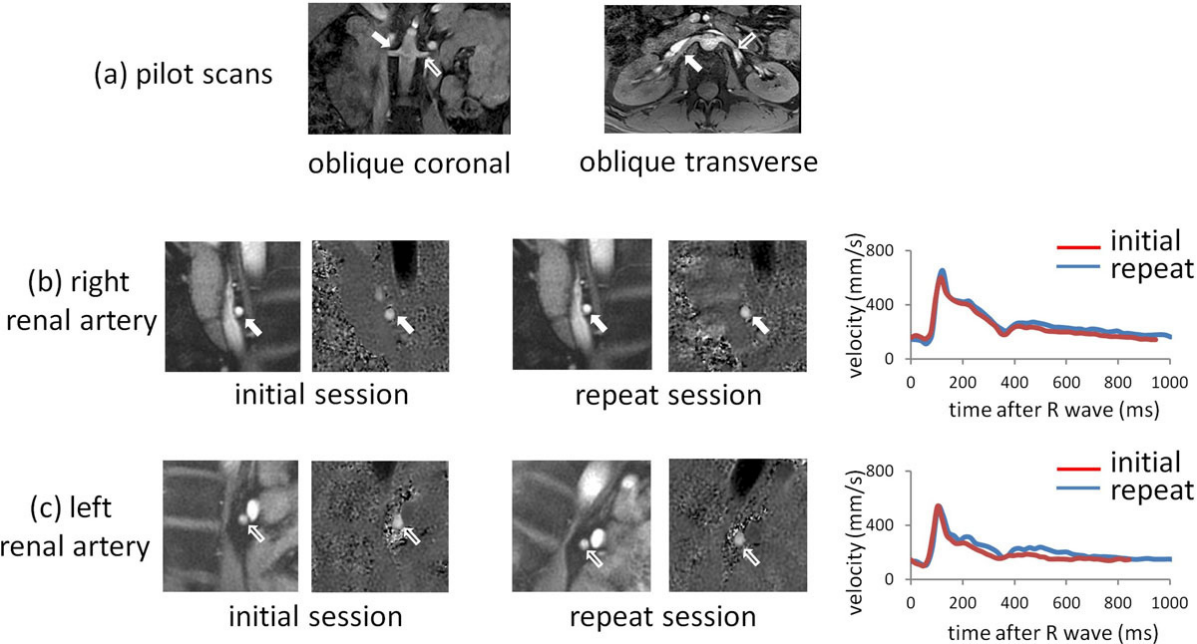
Example pilot scans showing the proximal renal arteries (a) together with systolic magnitude images and velocity maps in the initial and repeat scanning sessions and the temporal patterns of flow velocity through the cardiac cycle for the proximal right (solid arrows) (b) and left (open arrows) (c) renal arteries respectively.

**Figure 2:**
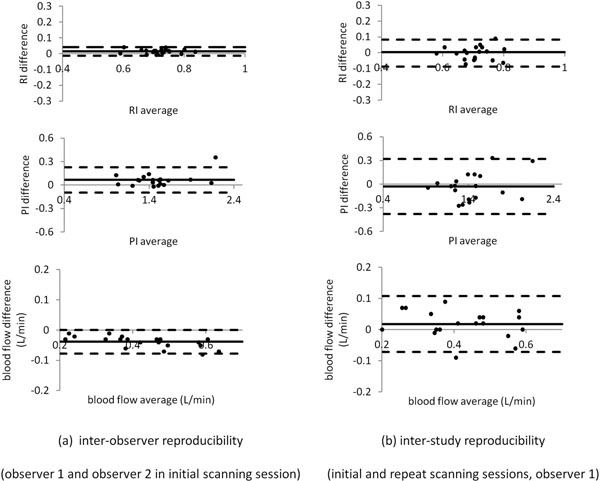
Inter-observer (a) and inter-study (b) Bland Altman plots of RI (resistive index), PI (pulsatility index) and RABF (renal artery blood flow) per kidney in 10 healthy volunteers. For each plot, the horizontal lines show the mean difference and the 95% limits of agreement (mean +/- 2SD) between observers (a) or between scanning sessions (b).

Figure 1 shows example data. RI, PI and RABF per kidney were 0.71+/- 0.06, 1.47 +/- 0.29 and 413 +/- 122 ml/min respectively. Inter-observer and inter-study Bland Alman plots are shown in Figure 2. The inter-study reproducibilities were: RI -0.00 +/- 0.04 , PI -0.03 +/- 0.17, and RABF per kidney 17.9 +/- 44.8 ml/min. Inter-study ICCs (observers 1 and 2 respectively) were 0.87 and 0.86 (RI), 0.92 and 0.93 (PI) and 0.96 and 0.95 (RABF). The effect of background phase correction was negligible (<2% for each parameter).

## Conclusions

High temporal resolution breath-hold spiral phase velocity mapping allows reproducible assessment of renal pulsatility indices and RABF. We conclude that this technique is suitable for studying temporal flow velocity patterns in the renal arteries.

## Funding

N/A.

